# Reactions to learning a “not elevated” amyloid PET result in a preclinical Alzheimer’s disease trial

**DOI:** 10.1186/s13195-018-0452-1

**Published:** 2018-12-22

**Authors:** Joshua D. Grill, Chelsea G. Cox, Kristin Harkins, Jason Karlawish

**Affiliations:** 1 Institute for Memory Impairments and Neurological Disorders, University of California, Irvine, 3204 Biological Sciences III, Irvine, CA USA; 2 Institute for Clinical and Translational Science, University of California, Irvine, Irvine, CA USA; 3 Department of Psychiatry and Human Behavior, University of California, Irvine, Irvine, CA USA; 4 Department of Neurobiology and Behavior, University of California, Irvine, Irvine, CA USA; 5 Penn Memory Center, University of Pennsylvania, Philadelphia, PA USA; 6 Department of Medicine, University of Pennsylvania, Philadelphia, PA USA; 7 Department of Medical Ethics and Health Policy, University of Pennsylvania, Philadelphia, PA USA; 8 Department of Neurology, University of Pennsylvania, Philadelphia, PA USA

**Keywords:** Disclosure, Preclinical, Asymptomatic, Alzheimer’s disease, Prevention

## Abstract

**Background:**

The experiences of biomarker-ineligible cognitively normal persons can inform trial conduct and the translation of preclinical Alzheimer’s disease (AD) into clinical practice.

**Methods:**

We interviewed 33 persons whose “not elevated” brain amyloid imaging biomarker result made them ineligible for a preclinical AD trial.

**Results:**

Most participants (*n* = 17) reported being informed that they did not demonstrate adequately elevated amyloid to qualify, whereas some (*n* = 14) reported being told they had no amyloid or plaques. Relief (*n* = 17) and disappointment related to not being able to participate (*n* = 12) were the most common reactions to results. Nearly all participants would have made healthy lifestyle changes if they had received an “elevated” result, would have another scan, and would participate in another AD prevention trial.

**Conclusions:**

Although some participants may misconstrue results, disclosure of a “not elevated” amyloid result in the research setting causes little behavior change; willingness to participate in AD research remains.

## Background

Increasing evidence supports a hypothesis that dementia due to Alzheimer’s disease (AD) is the end result of a long pathophysiological process that begins years before symptoms [1]. This asymptomatic or preclinical stage offers an opportunity to test disease-modifying therapies to delay the onset of cognitive and behavioral symptoms due to AD [2, 3].

To achieve feasibility, clinical trials in preclinical AD enroll and randomize to drug or placebo only persons meeting specific biomarker criteria [4]. Few data are available as yet that are related to the safety [5, 6] and impact [7] of biomarker disclosure in cognitively normal persons, and available reports have understandably focused on reactions among participants who qualify to enroll [8–11].

Disclosure of biomarker results in the clinical trial setting most frequently involves communication of negative results, such as not elevated brain amyloid and consequent ineligibility. Potential negative implications of this disclosure include misunderstanding and reduced motivation for optimal health behaviors [12–14]. The impact on willingness to participate in future trials is unknown. To explore the implications of communicating negative biomarker results to cognitively normal individuals, we performed telephone interviews with participants who screen failed for a preclinical AD trial due to inadequate brain amyloid deposition, as demonstrated through amyloid positron emission tomography (PET).

## Methods

### Participants

We interviewed individuals recruited to the Anti-Amyloid treatment in Asymptomatic AD (A4) study [15] at a single site. The A4 study enrolled individuals aged 65–85 years who were cognitively unimpaired (Mini Mental State Examination score 25 to 30, global Clinical Dementia Rating of 0, Logical Memory II score of 6 to 18) and had “elevated” brain amyloid as measured by amyloid PET. Eligible participants were randomized to receive the monoclonal antibody against amyloid beta, solanezumab, or placebo. Criteria for the current study were having screen failed for the A4 study due to a “not elevated” amyloid PET scan. We excluded those enrolled in the Longitudinal Evaluation of Amyloid Risk and Neurodegeneration (LEARN) study (*n* = 4), a prospective cohort study that follows a subset of participants who screen failed from A4 due to a not elevated amyloid PET result.

### Disclosure process

In the A4 study, all participants underwent a systematic process to disclose amyloid PET results [16]. The process mandates that disclosure be performed by a trained and qualified investigator, that education be provided at consent, that PET imaging be performed on a separate day from consent and disclosure to ensure participants have the opportunity to change their minds, and that the safety of disclosure be monitored before and after imaging through standardized assessments of depression, anxiety, suicidality, and distress. Results are disclosed as “elevated” or “not elevated” amyloid.

An educational study brochure is used as a tool accompanying the disclosure process. The brochure, informed consent, and disclosure process emphasize that amyloid imaging does not equate to a diagnosis of AD, that quantitative information on the risk and timing of future cognitive impairment and AD dementia among those with elevated amyloid is not known, and that individuals with a “not elevated” scan result could someday develop elevated amyloid or AD dementia.

### Data collection

An interview guide was developed that was based on the experiences of the investigative team in performing the disclosure process and the literature related to the potential positive and adverse consequences of biomarker disclosure [12, 13, 17–20]. Interviews included both forced choice and open-ended questions. Topics included expectations for the scan, impression of the disclosed PET result, meaning of the PET result, and what it would have meant to receive an elevated result. We used the Impact of Events Scale (IES) to assess distress associated with disclosure [21]. Participants were asked whether and why their study partner accompanied them to the disclosure visit. We asked participants with whom they subsequently shared their PET results. We assessed whether participants had made changes in health and planning behaviors since screening, including diet, exercise, prescription medications, vitamins or supplements, long-term care insurance, and advance healthcare directives. If participants responded affirmatively, we inquired about the types of changes that were made. If participants responded negatively, we confirmed that the factor was “exactly the same as it was before screening for the A4 study.” We next asked whether participants would have made changes in each factor if they had received an elevated PET result. Likert scales were used to assess participants’ willingness to repeat PET imaging and to enroll in another trial.

### Data analyses

Quantitative data were entered into a Research Electronic Data Capture (REDCap) database and summarized with descriptive statistics. We calculated the Pearson correlation coefficient to examine for a relationship between IES score and time since disclosure. We used Grubbs’ test to assess for outliers.

Interviews were recorded, transcribed, and entered into qualitative analysis software (NVivo version 11.0; QSR International, Melbourne, Australia). Two of us (JDG and CGC) reviewed a subset of transcripts to identify preliminary themes and engaged in a consensus-building exercise to arrive at themes for interview coding. We double-coded interviews, adjusting themes as necessary and intermittently assessing interrater consistency. In the event of a Cohen κ coefficient for reliability < 0.8, interviews were reviewed for coding consistency, and consensus was achieved. At the conclusion of data coding, we performed a final consensus exercise to ensure consistency in participants coded for each given qualitative theme. The final Cohen κ was > 0.9. For each theme, we report the frequency count of codes within that theme.

### Ethics

The University of California Irvine Institutional Review Board approved this study. Informed consent was performed telephonically and was acknowledged in writing by the investigator performing the study interviews.

## Results

### Participants

We interviewed 33 of 43 (77%) individuals eligible for this study. Seven individuals declined participation, and we were unable to reach three individuals after three attempts to contact them via telephone. Among those who declined, three were not interested, two were too busy, one had a family emergency, and one had a recent stroke. Participants interviewed were mostly white and highly educated, and about half reported a family history of dementia (Table 1). Interviews were performed between October 25, 2017, and December 12, 2017. The range of time between PET disclosure and study interview was 3–30 months (median, 20 months).

**Table 1:** Participant characteristics (*n* = 33)

Characteristics	Data
Female, *n* (%)	25 (76)
Non-Hispanic white, *n* (%)	25 (76)
Age, years, mean (SD) [range]	73.6 (5.3) [65–83]
Education, years, mean (SD) [range]	16.9 (2.2) [13–22]
Months between PET disclosure and interview, median [range]	20.0 [3–30]
IES score at time of interview, mean (SD) [range]	3.1 (6.2) [0–33]
Family history of dementia, *n* (%)	17 (52)
Study partner type, *n* (%)	
• Spouse	20 (61)
• Friend/companion	6 (18)
• Adult child	5 (15)
• Other	2 (6)

*IES* Impact of Events Scale

### Meaning and impressions of PET results

Table 2 summarizes the types of responses participants gave when asked to describe their PET results, as disclosed by the A4 research team. The large majority of participants acknowledged that their results made them ineligible to continue in the A4 study. Just over half (*n* = 17) indicated that their amyloid result was “not elevated” or “not enough plaque.” Just under half (*n* = 14) reported being told that they had “no amyloid,” “no plaques,” or “clear scans.” For example, one 83-year-old female stated, “*Um, in my own words I understood that I did not have any amyloids and my brain was clear. And that’s all I wanted to hear.*”

**Table 2:** Impression of disclosed amyloid positron emission tomography results

Theme/example quotes	*N*
The scan made the participant ineligible to enroll • “All I was told was that I was not eligible to move into the test.… I wasn’t told if I have the amyloid or not. I was just told that I wasn’t eligible, which I interpreted that to mean that if I do have it, it wasn’t at the level necessary to move into the test.” (67-year-old male) • “What I just came away with that um, yes I have plaque but not, I didn’t qualify for the test. Now what percentage of plaque I have, I don’t, you know, I didn’t put that in my head to remember that, you know, that I need to know that I have X percentage of plaques. So, I just know that I was told that I didn’t qualify and that’s what I came away with.” (80-year-old female)	26
The scan failed to demonstrate adequate plaque burden • “The um level of, uh, amyloid plaque found in my brain was below the cutoff level for the study. They said it was not elevated.” (79-year-old female) • “They were looking for people with a certain number of amyloid things in the brain, amyloids or whatever they were. And they wanted people with just enough, not too many and not too few. And I guess the results of mine were that I didn’t have quite enough yet.” (72-year-old male)	17
The scan demonstrated the absence of plaques • “They just said I didn’t have any evidence of plaques or any other abnormalities.” (72-year-old male) • “Bottom line is I did not have the pathology for Alzheimer’s. That’s all I know.” (78-year-old female)	14
The scan result could change in the future • “Oh, and they also said just because I don’t have the amyloids in my brain, that doesn’t mean that I won’t get Alzheimer’s.” (67-year-old female) • “It meant that at least that day, when they did the PET scan, I didn’t have amyloids. It was nice to hear, doesn’t mean I won’t get them, but at least I don’t have them on that day. No guarantees.” (69-year-old female)	8
The scan failed to demonstrate adequate tangles • “Apparently, I had insufficient plaques and tangles to be considered a viable member of the study.” (66-year-old female) • “They uh did not find um, evidence of any of the plaques or tangles that are associated with Alzheimer’s. That’s all I remember.” (68-year-old female)	2
Other • “Um, the results were that I was not in the area where the um, I was not in the amyloid area.” (72-year-old female)	1

Eight participants acknowledged that their scan results or risk for dementia could change in the future. Two participants indicated that they had been told they had inadequate neurofibrillary tangles to participate.

### Reactions to PET results

The most common reaction to learning of a not elevated PET result was relief (*n* = 17). Twelve participants indicated that they were disappointed that they were not eligible to continue in the study (Table 3). One 67-year-old female stated, “*Well, it was a relief on one hand, uh, to know that I didn’t have that condition. And then it was a little bit of a disappointment that I couldn’t be in the study. So, I guess it was more of a relief, but then I still wanted to know what I could do to help with finding a cure for the disease.*”

**Table 3:** Reactions to amyloid positron emission tomography results

Theme/example quotes	*N*
Learning the result caused relief • “Well, actually it was a relief really because they said it would be unlikely that I would get Alzheimer’s and given the fact that I have several members in my family that have that, it was actually a relief, because I thought, oh well I don’t have to deal with that one, it will be something else that I’ll die from probably.” (78-year-old female) • “It was a big relief to me. It really made my day and it’s making my day all today, every day, to know that I don’t have that. That if I have it, it’s not that bad or that serious or anything like that, you know, I’m pretty okay. Pretty much.” (77-year-old female)	17
Learning the result caused disappointment that the participant could not continue in the study • “Well I didn’t get to go through any more tests. The tests seemed to be, at times, fun. So, you pulled the joy plug.” (80-year-old male) • “I guess one feeling was disappointment because we couldn’t continue. Um, we had become invested in it to some extent. It became something that we were really interested in, um…, so I felt disappointed that we weren’t going to continue.” (74-year-old female)	12
The result was unexpected or caused surprise • “Um, I assumed because of my twin brother that I would have at least some elevated levels. I was quite surprised to the point of tears when [the study physician] told me.” (77-year-old male) • “I was very surprised because I really expected something different. I expected that there was already something happening because it started early in my mother. So I was pleasantly surprised, but um, it was excellent.” (69-year-old female)	12
The result meant that the participant was at relatively lower risk for AD • “But it also meant that I’m not about to experience Alzheimer’s disease any time soon. Um, possibly later I could, uh, but um, I also was glad that I could tell my children that their level of risk is not too high.” (79-year-old female) • “It meant that um … I could not be in the study because I may not have, I have a lesser chance of having Alzheimer’s disease, but that doesn’t mean that I won’t have it.” (78-year-old female)	11
The result was expected • “Well, it just kind of validated what I probably already thought because I know that Alzheimer’s is a hereditary disease and since neither one of my parents had it, any sense of it, they were both sharp as a tack until the day that they died. Um, so that was not a surprise, but it was still interesting to know and comforting to know that um, I didn’t have that pathology.” (78-year-old female) • “I feel I’m functioning pretty well, so I didn’t expect any abnormalities. Um, also, my family history is one that, um, nobody has had Alzheimer’s in my side of the family.” (72-year-old male)	8
The result meant that the participant is not at risk for AD • “It meant that I probably will not have Alzheimer’s disease.” (70-year-old female) • “I mean at least for as much as research has gotten to the point of thinking that plaques and tangles may be important, um, you know, then since I don’t have them yet or at least not in large quantity, that would suggest that at least one form of dementia is not in my future.” (66-year-old female)	4
The participant desired to learn more information about the scan result, beyond the information disclosed • “But then I asked well, the level, I mean you got a grading of the level, what is it? He says, well we can’t tell you that. In fact, they don’t even know, the people who were doing the research don’t. I think we were told at the beginning, but anyway, so it’s, it’s, that doesn’t matter. I mean, it would’ve been nice if I’d known what it was, but I knew going in that they wouldn’t give me that number anyway.” (77-year-old male) • “Um, well it didn’t mean much. We just knew that we didn’t have a severe problem, and that’s reassuring. Um, but, you know we could you know, have a 9% plaque buildup and we wouldn’t know that sort of thing.” (78-year-old male)	2
The result reinforced the participant’s choices related to lifestyle • “So for me it reinforced my belief that whatever it is that I’m continuing to do in my life, or whatever I’m doing in terms of trying to take care of my brain, I should continue to do it as much as possible.” (67-year-old male)	1

Eleven participants indicated that they felt their risk for AD was reduced on the basis of their PET results; four indicated that they had no risk for AD. Twelve participants expected their PET results to be elevated, and eight expected the results they received; thirteen had no expectations. Two participants indicated a desire to learn more quantitative information about their PET results.

### Distress due to PET results

IES scores of most participants (*n* = 31) at the time of interview were indicative of subclinical distress related to learning their PET results (Table 1). One score indicated mild distress, and one indicated moderate distress. There was a weak negative correlation between time since disclosure and IES score (*r* = − 0.17). Grubb’ test indicated the moderate distress score was an outlier (*p* < 0.05). After this case was removed, there was no relationship between time since disclosure and IES score (*r* = − 0.07).

### Role of study partner

When asked about the involvement of the study partner in the disclosure visit, 14 participants indicated that their partner was present, whereas 19 indicated they received their results alone. When asked why they received their results alone, 12 indicated that the involvement of the partner was not necessary, 10 said that involving the partner in the disclosure visit would have been inconvenient, and 8 said that they were unaware that the partner could attend the disclosure visit. For example, a 70-year-old female reported, “*I didn’t know that was an option. Otherwise, I would’ve included him. I wasn’t advised that I could bring someone. I don’t remember being advised. Maybe I was but I don’t remember it*.” Four participants who had their partner present reported that the partner provided needed support during the disclosure visit. When the same individual was asked if she would have preferred that her partner be present for the disclosure visit, she responded, “*Yes, I think so. Just to have a supportive person there.*”

### Sharing of PET results

Every participant shared his or her amyloid PET result with more than one other person, most commonly spouses (*n* = 29), adult children (*n* = 28), and friends (*n* = 28). Nineteen participants shared their results with their primary care physician. The reasons for sharing were to keep others in their lives informed about their health (*n* = 21), as a means to encourage others to participate in research studies (*n* = 9), to confirm their cognitive health to others (*n* = 8), and to provide relief to others in their lives, primarily family members (*n* = 5).

### Health behaviors

None of the participants acknowledged making lifestyle changes deemed negative to health (e.g., reduced exercise or poorer diet) since learning their PET results. A few (*n* = 5) indicated that they had made positive changes in health behaviors as a result of heightened sensitivity to brain health. Nearly all participants indicated that they would have made a lifestyle change had their scan results been elevated, including changing their diet (*n* = 25), exercise (*n* = 24), prescription medications (*n* = 22), and vitamins or supplements (*n* = 24).

### Planning behaviors

Half of participants already had long-term care insurance (*n* = 16), and nearly all had an advance directive in place (*n* = 32). Seven participants lacking long-term care insurance indicated they would have made a change had their results been elevated. The participant without an advance directive indicated she would have established one had her result been elevated. Of participants with advance directives, six would have made changes had their scan results been elevated. Though most did not describe a specific change, two indicated they would have altered choices related to life-sustaining therapy and do-not-resuscitate orders.

### Future trial participation

When asked the likelihood of participating in another AD prevention trial, some participants indicated that they would be very (*n* = 21) or somewhat (*n* = 12) likely to do so. The comments by one 66-year-old female are representative of those of the other participants: “*Very likely. I had a good experience with as far as I got with the first one. So, another opportunity to do something slightly different would uh, I’d be very happy to participate.*”

Nearly every participant (*n* = 30) indicated that they would be very (*n* = 21) or somewhat (*n* = 9) likely to undergo another amyloid PET scan if given the opportunity. Table 4 outlines themes of participant reasons for and against undergoing repeat amyloid imaging. Reasons to repeat testing outnumbered reasons not to and included to assess for change in amyloid status, to help with research, to learn AD risk, and curiosity. Reasons not to undergo repeat testing included negative experiences or discomfort with imaging and feeling that additional testing was unnecessary.

**Table 4:** Desire to repeat amyloid positron emission tomography scan

Themes that endorsed undergoing repeat amyloid PET/example quotes	*N*
To assess potential change in the scan result • “Because like they told me at the end that there wasn’t any assurance that just because I didn’t have enough amyloid then that it didn’t get out of control between then and now or that I was progressing and didn’t know it.” (82-year-old female) • “Well, because, you know, when they say at this time, you know you kind of wonder well, maybe I just didn’t have it yet but could be getting it later, you know. So it would be interesting to uh, find out if in fact I was getting it, but just later than my sister.” (72-year-old female)	12
To help advance research • “Again, I would like to help out with any tests I could do that would help find a cure for Alzheimer’s.” (73-year-old female) • “Um, I didn’t have any problems with the procedure, I thought it was an interesting thing to do, and um, again, you know, if it’s a data point that helps with research, why not?” (66-year-old female)	11
To learn updated risk for AD • “Because I’d rather know than not know … whether there is at least some indication of susceptibility to uh, Alzheimer’s or another dementia.” (77-year-old male) • *“*Oh, that’s easy. Very likely. I like to know. Because forewarned is forearmed. In other words, if I found out that I had uh, say it was just a study to find out oh, how many people have it, I’d be interested in knowing because that tells me, gee, uh, you might be at a higher risk, so that tells me, I had better do something about it, or at least I should watch it.” (77-year-old male)	8
Curiosity • “Oh, I’m curious to see whether I’m going to develop them or not. I’m just, just a general curiosity, too, and it’s something to do. I’m retired, so.” (72-year-old male) • “Well because maybe, it’s not so much I have to know. It would be nice to know.” (69-year-old female)	7
Themes that did not endorse undergoing repeat amyloid PET/example quotes	
Due to a negative experience or safety concerns • “Because I’ve already had one, and I didn’t enjoy that PET scan. I have claustrophobia, and that was all I could do to take care of that one.” (78-year-old female) • “I would say, why don’t you use this recent PET scan we already had…, because I don’t like radiation, especially on top of even more radiation…. However, I might rethink that if it’s a good study.” (78-year-old male)	5
Repeat imaging is unnecessary • “Well probably unlikely because if I was negative a year ago and I haven’t seen any issues, I don’t think anything would be popping up in a year.” (67-year-old male) • “I don’t think I feel the need to have one now. I might reach a point in the future that I would be interested or willing to have another. I think again, if I reached a point where I thought my memory was uh, that I was really concerned about it, I would probably, and I was interested in getting a diagnosis or um, knowing some heightened risk situation, I’d probably follow up with it again.” (68-year-old female)	4

## Discussion

Clinical trials frequently disclose AD biomarker results to cognitively normal individuals. Disclosure processes focus on educating potential subjects about what is known and not known about amyloid imaging, minimizing negative reactions, and ensuring the safety of those who are informed that AD biomarkers are present [16, 22]. Better understanding of the impact of learning a “negative” result is needed, given that this will be the most common type of information disclosed in future trials and ultimately in large public health screening campaigns when effective therapies are developed. This study of individuals who learned they did not have an elevated amyloid result shows how trial participants understood, reacted to, and used this information as well as whether they would participate in another study.

A majority of participants reported that they were informed they had insufficient amyloid to qualify for the trial, but nearly half of participants described a result that was different from the A4 disclosure materials (Table 2). These participants reported being told they had no amyloid or plaques in the brain. Similarly, only a few participants acknowledged that the results of their amyloid PET scan could change over time. These findings may reinforce the need to emphasize before and after disclosure that an amyloid PET result can change.

Other potential improvements to the disclosure process include a greater focus on who can be present. Some participants were unaware that their study partner could join them at the disclosure visit. The role of the study partner in this process, and in preclinical AD trials more broadly, remains an area of active study [23, 24]. Although most participants in this study felt that the presence of the partner during disclosure was unnecessary, a smaller portion felt that the partner provided needed support. Sixteen participants indicated that they would have felt concern or distress had they received an elevated result (data not shown). In this scenario, support from a study partner might have been valuable to them. Optimizing the process of disclosure, including ensuring that participants have adequate support to cope with results, will be necessary to ensure the safety and satisfaction of future trial participants.

Only two participants voiced a desire to learn more information about their disclosed result (Table 3). This is in contrast to a recent study of individuals with elevated amyloid in which 40% voiced displeasure with not receiving more information about their PET result [7]. Quantitative biomarker results are not provided in preclinical AD trials, in part because the optimal thresholds for identifying those at greatest risk for cognitive decline are uncertain [25, 26]. Given this, the criteria for biomarker eligibility may change in future preclinical AD trials. Our results indicate that people who fail screening on the basis of biomarker test results will be likely to participate in additional trials if invited. In fact, most were interested in undergoing repeat amyloid imaging, for reasons including curiosity, desire to learn about changes in risk for AD dementia, and desire to contribute to research.

Ongoing research seeks to assess the safety of AD biomarker disclosure, with preliminary studies indicating disclosure does not cause clinical depression or anxiety [6]. Not surprisingly, the majority of participants in this study did not experience distress in response to learning their biomarker results. One participant, however, did experience intrusive thoughts related to the disclosure process and scored in the moderate range on the IES. Specifically, this participant communicated concerns regarding not asking for or receiving enough information about the result and worry that the result could change in the future. It is possible her reaction resulted from subjective cognitive complaints left unexplained. This would be consistent with previously reported feelings of ambiguity and frustration among participants with mild cognitive impairment (MCI) in response to a test that fails to provide additional diagnostic information when symptoms are present [27, 28]. Nevertheless, the most common reaction to the disclosed information in this sample, as in studies of MCI [27], was relief. Notably, the second most common reaction was disappointment in not being able to contribute to the study.

Few people used their amyloid PET scan results to change behavior. None used the disclosed information to justify adoption of unhealthy lifestyle choices. A few developed a heightened sensitivity to issues of brain health and initiated or improved behaviors, such as eating a healthier diet or exercising more. Most, however, made no lifestyle changes. Nearly all speculated that they would have changed their behavior had their result shown elevated amyloid. These preliminary results are important to future screening campaigns, suggesting that in the setting of a substantive education program, the risk for people using screening information to justify unhealthy lifestyle choices (e.g., greater consumption of high-fat or high cholesterol foods, reduced exercise), which could increase risk for future cognitive impairment [29], may be low. But people may also be less motivated to adopt healthy behaviors. Based on current understanding and available prevention strategies [30], the recommendations for lifestyle and medical choices are no different for individuals who do or do not demonstrate elevated amyloid. All individuals undergoing screening should be educated and empowered to adopt risk-reducing lifestyle strategies.

Learning that they have increased risk of developing AD dementia due to elevated amyloid may cause some individuals to set up advance healthcare directives and other medicolegal arrangements such as living wills [14]. Reduced motivation to complete such arrangements due to biomarker outcomes could have negative implications, especially for individuals who are at high risk for other causes of late-life disability, such as stroke or myocardial infarction, even if their risk for AD is relatively lower. A high proportion of participants in this study had advance healthcare directives and long-term care insurance. Seven of 17 participants without long-term care insurance indicated they would have established a policy had they received an elevated PET result. The one participant who did not have an advance healthcare directive indicated that she would have established one had her result been elevated. Collectively, these findings indicate the need for future trials to further emphasize medicolegal recommendations for study participants, regardless of the outcome of biomarker testing.

Limitations of this study include that participants were recruited from a single site, where a single investigator primarily performed the disclosure process. The homogenous sample may not be representative of participant reactions to negative amyloid imaging results delivered across a large multisite trial by multiple investigators, let alone outside the research setting. Assessment of site heterogeneity in disclosure processes and effects on participant responses will be an important area of future research. Participants were predominantly white, female, and highly educated, potentially limiting generalizability to different groups. Whether the ten eligible individuals who we were unable to reach or who declined participation responded differently to learning their amyloid PET results than those who did participate is unknown. There was a wide range in the durations between disclosure and data collection. This could have introduced a confounding variable if, for example, emotional reactions to learning a PET result change over time. The variability in time since disclosure may also have caused heterogeneity in the accuracy of reported experiences.

## Conclusions

These results suggest that the disclosure process in the A4 study was effective and safe in ineligible participants, though important opportunities to improve the process may exist. Disclosure did not result in adoption of unhealthy behaviors among participants; however, most indicated that there were risk-reducing strategies that they would have implemented had they received an elevated result. Communicating the need to adopt such strategies to all participants will be important in future preclinical AD trials, as well as in future public health campaigns that aim to identify appropriate candidates for disease-delaying therapies.
